# Advances in FPGA-Based Laser Frequency Stabilization Techniques

**DOI:** 10.3390/mi17070838

**Published:** 2026-07-14

**Authors:** Zhilin Yan, Wenqiang Fan, Longjie Zhang, Wanxiao Gao, Cunwei Zhang, Jiaming Zhang, Tie Li, Yancheng Guo, Yulei Wang, Zhiwei Lu, Qiunan Yang, Zhenxu Bai

**Affiliations:** 1 Center for Advanced Laser Technology, Hebei University of Technology, Tianjin 300401, China; 2 Hebei Key Laboratory of Advanced Laser Technology and Equipment, Tianjin 300401, China; 3 Science and Technology on Particle Transport and Separation Laboratory, China National Nuclear Corporation, Tianjin 300180, China

**Keywords:** laser frequency stabilization, FPGA, digital feedback control, Pound–Drever–Hall technique

## Abstract

Laser frequency stabilization underpins precision metrology, optical atomic clocks, quantum optics, and laser spectroscopy. In recent years, field-programmable gate arrays (FPGAs) have become attractive for this task because signal generation, phase-sensitive detection, digital filtering, feedback control, lock monitoring, and automatic re-locking can be integrated on compact and reconfigurable platforms. This review examines recent progress in FPGA-based laser frequency stabilization from four linked perspectives: stabilization principles, digital implementation, system architecture, and intelligent control. We first summarize representative error-signal generation methods, including Pound–Drever–Hall locking, saturation absorption spectroscopy, frequency modulation spectroscopy, and modulation transfer spectroscopy. We then discuss the FPGA functions that determine practical performance, such as data acquisition, direct digital synthesis, digital demodulation, proportional-integral-derivative (PID)/infinite impulse response (IIR) filtering, latency management, and lock-state monitoring. Mixed-signal, all-digital, distributed, and machine-learning-assisted systems are compared to show how bandwidth, latency, stability, integration, cost, and automation are balanced in different designs. This review closes by identifying remaining challenges in analog-to-digital converter/digital-to-analog converter (ADC/DAC) resolution, converter noise, loop latency, actuator bandwidth, long-term robustness, and algorithm portability, and by outlining future directions toward low-latency, software-defined, and intelligent stabilization platforms.

## 1. Introduction

Owing to its high monochromaticity, strong directionality, high coherence, and high brightness, the laser has played a central role in the development of modern science and technology. In precision sensing and measurement, lasers serve both as carriers of information and as essential tools for the high-sensitivity detection of physical quantities [1]. Laser technology is widely used in atomic and molecular physics, optical communications [2,3], precision machining, and biomedical applications [4,5].

Laser frequency stability is a key parameter that sets the performance level and practical limits of laser-based systems [6,7], particularly for single-frequency lasers [8,9,10,11,12], where narrow linewidth and high coherence are essential for high-resolution sensing applications. Such lasers are sensitive to environmental perturbations, including acoustic noise and temperature variations, which readily induce frequency drift and linewidth broadening. Frequency stabilization techniques are therefore required to maintain stable operation and preserve spectral purity [13]. These techniques are central to a wide range of applications, including precision spectroscopy [14], atomic clocks [15,16,17], gravitational wave detection [18,19], and quantum information science [20].

**Figure 1:**
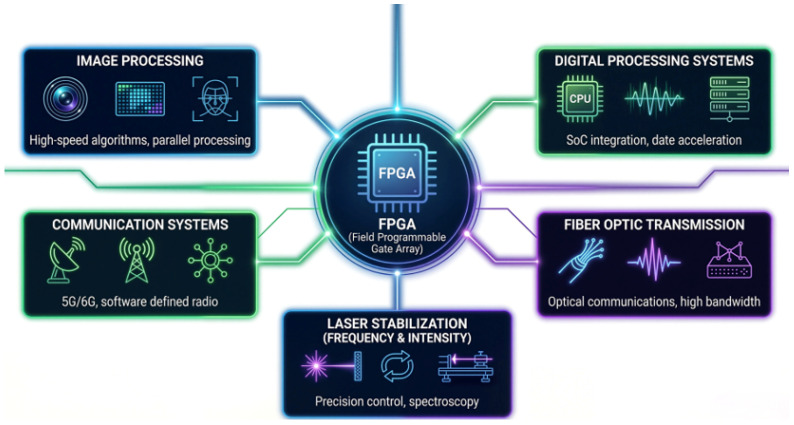
The application of FPGA.

Traditional laser frequency stabilization systems are typically implemented using analog circuits, which suffer from drawbacks such as bulky size, complex control, susceptibility to temperature drift, and low integration. A field-programmable gate array (FPGA) is a semiconductor device capable of functional reconfiguration through hardware description languages. It enables the implementation of customized circuits at the hardware level, and its inherent parallel execution architecture demonstrates outstanding performance when processing complex digital logic. FPGAs have gained widespread application in various fields of industrial control. As shown in Figure 1, FPGA-based implementations have been widely applied in a range of systems, including image processing [21], digital processing systems [22], communications [23], fiber optic transmission systems [24], and laser frequency and intensity stabilization systems [25,26]. Recent advances in FPGA technology, leveraging its parallel processing capabilities, high-speed data handling, programmability, and high integration density, have demonstrated significant potential in laser frequency stabilization applications. Numerous research groups around the world have applied FPGAs to laser frequency stabilization [27,28].

This paper reviews the application of FPGAs in laser frequency stabilization. We summarize recent progress, outline the underlying technical principles, compare representative implementation approaches, and discuss key experimental demonstrations. Finally, we highlight current challenges and future directions toward more integrated, fully digital, and increasingly automated stabilization architectures.

## 2. Active Frequency Stabilization Methods Using FPGA

Active frequency stabilization methods are primarily categorized into two types: those based on optical resonators and those based on atomic or molecular transition frequencies. The former is represented by Pound–Drever–Hall (PDH) technology, while the latter includes saturated absorption frequency stabilization and its related modulated spectroscopy methods. With the advancement of digital electronics, FPGAs have provided digital solutions for frequency stabilization. The following sections will introduce the principles and procedures of typical active frequency stabilization methods.

### 2.1. Pound–Drever–Hall Frequency Stabilization Technique

**Figure 2:**
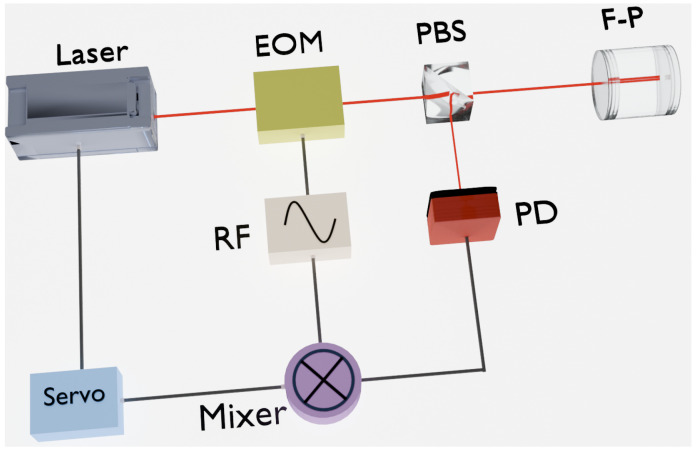
PDH frequency stabilization technology. EOM: electro-optic modulator; RF: radio frequency; PD: photodetector.

The core principle of the PDH frequency stabilization technique [29,30,31,32,33,34] is to use the resonant frequency of an optical resonator as a stable frequency reference, as illustrated in Figure 2. In this method, the laser beam is phase-modulated by an electro-optic modulator (EOM) before entering the optical resonator, generating two sidebands symmetrically distributed around the carrier. After interaction with the cavity, the reflected light containing the carrier and sideband components is detected by a photodetector and converted into an electrical signal. This signal is then demodulated with the original radio-frequency (RF) modulation signal to obtain a dispersive error signal that sensitively reflects the frequency deviation between the laser and the cavity resonance. After amplification and filtering, the error signal is sent to a servo controller, which regulates the laser frequency through a piezoelectric transducer (PZT) or current modulation, thereby forming a closed-loop feedback system that locks the laser frequency to the cavity resonance [35,36,37,38,39].

### 2.2. Laser Frequency Stabilization Based on Saturation Absorption Spectroscopy

In hot atomic (or molecular) gases, thermal motion of atoms (or molecules) causes spectral line broadening—known as Doppler broadening—rendering the line center frequency unsuitable as a direct frequency stabilization reference. To obtain narrow linewidth characteristics unaffected by Doppler broadening, two counter-propagating laser beams are employed: the stronger beam serves as the pump light, while the weaker beam functions as the probe light. Both beams interact with the atomic (or molecular) vapor. When the probe beam frequency matches the atomic transition frequency, both beams resonate simultaneously with atoms in the zero-velocity group. The pump beam selectively saturates these atoms, significantly weakening their absorption. This creates a narrow-linewidth saturated absorption feature (Lamb dip) at the center of the broadened absorption line. The center frequency of this saturation absorption feature represents the true transition frequency of the atom (or molecule), unaffected by the Doppler effect, serving as the reference frequency for frequency stabilization. In laser frequency stabilization techniques based on saturation absorption, techniques such as modulation or differential detection are typically required to extract an error signal with zero-crossing characteristics from the saturation absorption signal for use in closed-loop control. Currently prevalent saturation absorption-based frequency stabilization methods include direct saturation absorption spectroscopy (SAS) stabilization, frequency modulation spectroscopy (FMS) stabilization, and modulation transfer spectroscopy (MTS) stabilization. By leveraging the stable frequency reference provided by atomic spectral lines, the long-term stability of these methods has reached $10^{-8}$ to $10^{-14}$ [40,41,42,43].

#### 2.2.1. Direct Saturation Absorption Spectroscopy for Frequency Stabilization

**Figure 3:**
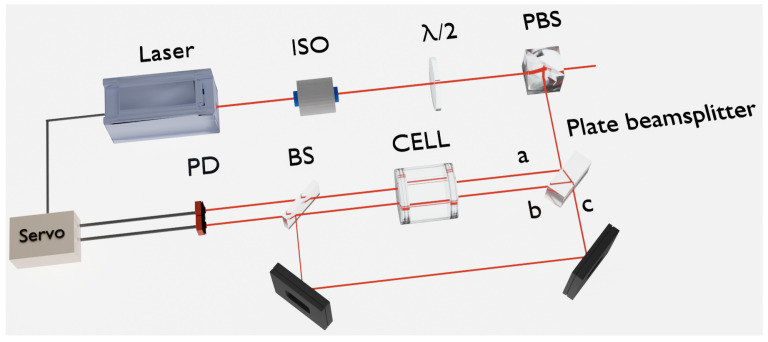
Direct saturation absorption spectroscopy for frequency stabilization (Path a: reference beam; Path b: probe beam; Path c: counter-propagating pump beam). ISO: optical isolator; PBS: polarizing beam splitter; BS: beam splitter; PD: photodetector.

The core principle of direct SAS frequency stabilization is to use the narrow linewidth feature in the saturation absorption spectrum as an absolute atomic or molecular frequency reference, as shown in Figure 3. In this scheme, the laser first passes through an optical isolator and a half-wave plate for polarization control, and is then divided by a polarizing beam splitter (PBS) into an output beam and a locking beam. The locking beam is further split into three beams: a reference beam carrying the Doppler-broadened background, a probe beam passing through the atomic or molecular absorption cell, and a counter-propagating pump beam. Inside the gas cell, the strong pump beam saturates the zero-velocity atoms or molecules, reducing the absorption of the probe beam and producing a narrow saturation absorption feature on the Doppler-broadened background. Near this feature, the probe-beam intensity varies with laser frequency and can be used to extract an error signal by differential or slope detection. After amplification and filtering, the error signal is sent to a servo controller, which regulates the laser frequency through a PZT or current modulation, thereby forming a closed-loop feedback system that locks the laser frequency to the absolute transition frequency of the atom or molecule [44].

#### 2.2.2. Frequency Modulation Spectroscopy for Frequency Stabilization

**Figure 4:**
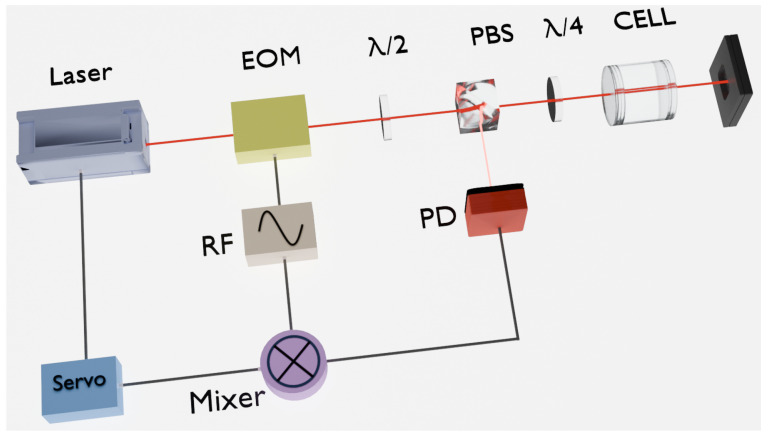
Frequency modulation spectroscopy for frequency stabilization. EOM: electro-optic modulator; PBS: polarizing beam splitter; PD: photodetector.

FMS frequency stabilization improves upon direct saturation absorption spectroscopy by introducing high-frequency phase modulation, as shown in Figure 4. In this method, the laser is phase-modulated by an electro-optic modulator (EOM) or by current modulation, generating two oppositely phased sidebands around the optical carrier. After polarization control, the modulated beam passes through the absorption cell and is reflected back to interact with the atomic medium again. Near the atomic transition frequency, absorption and dispersion in the vapor cell induce small amplitude or phase imbalances between the carrier and sidebands. The returned detection beam is separated by the PBS and detected by a photodetector, and the resulting electrical signal is demodulated with the original radio-frequency reference to extract a dispersive error signal with a steep slope near the spectral line center. By shifting the stabilization signal to a higher frequency band, FMS can suppress low-frequency background drift and 1/f noise. After amplification and filtering, the error signal is sent to a servo controller, which adjusts the laser frequency through a piezoelectric transducer (PZT) or current modulation, thereby locking the laser frequency to the atomic transition frequency.

#### 2.2.3. Modulation Transfer Spectroscopy for Frequency Stabilization

**Figure 5:**
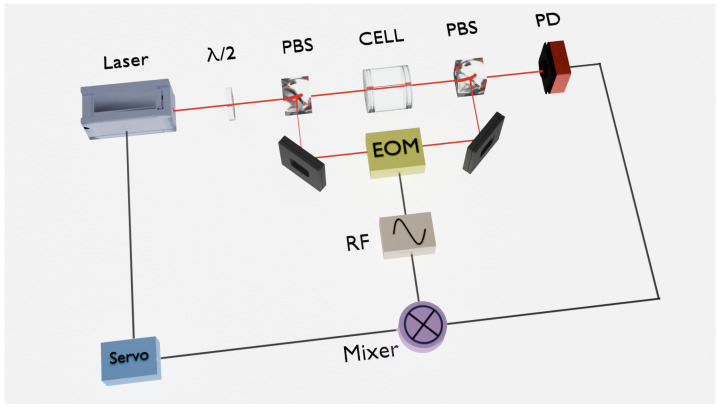
Frequency stabilization based on modulation transfer spectroscopy. EOM: electro-optic modulator; PBS: polarizing beam splitter; PD: photodetector.

MTS is a frequency stabilization technique based on nonlinear four-wave mixing, as shown in Figure 5 [45]. In this method, the laser beam is first polarization-controlled by a half-wave plate and then split by a PBS into a pump beam and an unmodulated probe beam. The pump beam is phase-modulated by an electro-optic modulator, generating oppositely phased sidebands around the optical carrier, while the probe beam directly passes through the atomic vapor cell in the opposite direction. Near atomic resonance, the modulated pump beam and the counter-propagating probe beam interact through nonlinear four-wave mixing, transferring the modulation information from the pump beam to the probe beam. This process generates a background-suppressed dispersive error signal with a steep zero-crossing slope while effectively reducing Doppler-broadened background and laser intensity noise. The transmitted probe beam is detected by a photodetector and converted into an electrical signal, which is then demodulated with the original modulation reference. After amplification and filtering, the resulting error signal is sent to a servo controller to regulate the laser frequency through a PZT or current modulation, thereby locking the laser frequency to the atomic transition frequency.

Figure 2, Figure 3, Figure 4 and Figure 5 should be read as schematic diagrams that explain the operating principles of representative laser frequency stabilization methods, not as fixed experimental configurations. For this reason, the laser and photodetector parameters are not specified as single values in the diagrams. In practice, the laser wavelength is chosen according to the optical-cavity resonance or the target atomic/molecular transition. Output power, free-running linewidth, tuning coefficient, and current or piezoelectric tuning bandwidth then have to be matched to the required error-signal slope and feedback bandwidth. The photodetector requires the same kind of matching: it should operate at the relevant wavelength and modulation frequency while providing sufficient responsivity, bandwidth, saturation power, and noise performance. In FPGA-based systems, its output amplitude and bandwidth also need to fit the ADC input range and sampling rate so that the digitized error signal retains an adequate signal-to-noise ratio. For the gas-cell-based schemes in Figure 3, Figure 4 and Figure 5, the cell represents an atomic or molecular frequency reference. The gas species is therefore selected according to the target transition and application wavelength; common examples include alkali-metal vapors such as Rb or Cs, iodine molecules, and other atomic or molecular gases. Gas species, temperature, pressure, cell length, optical power, and modulation depth can all influence linewidth, signal-to-noise ratio, background suppression, and long-term lock stability.

## 3. Implementation of Core FPGA Functions in Laser Frequency Stabilization

Conventional frequency stabilization systems typically rely on analog circuitry, including components such as signal generators, mixers, low-pass filters, and proportional-integral-derivative (PID) controllers. In contrast, the FPGA offers significant advantages, including parallel processing capability, high reconfigurability, and system integration [46,47,48]. As performance, integration density, and flexibility requirements in laser stabilization systems continue to grow, the FPGA is increasingly being employed to replace these analog modules, enabling fully digital implementations of laser frequency stabilization [49]. The FPGA performs several critical functions in laser frequency stabilization systems, including data acquisition and preprocessing, direct digital synthesis (DDS) and digital demodulation, digital PID control algorithm and intelligent state monitoring and system control.

### 3.1. Data Acquisition and Preprocessing

In laser frequency stabilization systems, real-time data acquisition is essential for frequency stabilization and feedback control. The system captures photodetector signals for amplification, filtering, and demodulation to generate corresponding laser feedback. Within an FPGA, digital filtering and demodulation extract an error signal representing the laser frequency deviation. These rapidly varying signals with wide dynamic ranges demand high sampling rates and excellent signal-to-noise ratios, making FPGA-based high-speed acquisition systems particularly advantageous.

Upon entering the FPGA, electrical signals undergo preprocessing. The FPGA first performs amplitude correction, bias elimination, and digital filtering on the raw acquired digital signals to suppress system noise. The preprocessed signals are then demodulated with the demodulation signals, filtered again to obtain the error signal, and subsequently fed into the PID controller for feedback control.

### 3.2. DDS and Digital Demodulation

In laser frequency stabilization, frequency scanning and modulation are essential for techniques such as generating radio-frequency sidebands in PDH stabilization or scanning the laser frequency across atomic or molecular absorption lines in saturation absorption spectroscopy. Traditionally implemented using analog electronics, these functions often suffer from limited linearity, coarse frequency resolution, and long-term instability. In contrast, FPGA-based DDS offers high precision, flexibility, and excellent stability, establishing it as a central component of modern digital frequency stabilization systems.

DDS is a signal generation technique that leverages digital signal processing to produce high-precision waveforms. It provides high frequency resolution, rapid frequency switching, and continuous phase output. Based on the sampling theorem, DDS operates by storing a sampled, digitized waveform in a lookup table, which is then sequentially read and reconstructed into an analog signal through a digital-to-analog converter (DAC). The fundamental DDS architecture comprises four key components: a phase accumulator, a waveform lookup table, a DAC, and a low-pass filter.

Digital demodulation is critical for extracting the error signal in laser frequency stabilization. After digitization with a high-speed ADC, the signal is processed synchronously inside the FPGA along with a DDS-generated local reference. Using embedded high-speed multipliers, the FPGA mixes the acquired photodetector signal with the reference to perform demodulation. The resulting signal contains a DC component corresponding to the error signal, along with high-frequency components at harmonics of the modulation frequency. A low-pass filter—typically implemented as a finite impulse response (FIR) or infinite impulse response (IIR) filter—removes these high-frequency terms to yield a clean error signal. This all-digital demodulation method avoids DC offset drift and other imperfections common in analog mixers, thereby improving the signal-to-noise ratio of the error signal and enhancing the long-term stability of the stabilization system.

The DDS module implemented via FPGA not only enables precise frequency and phase control [50] but also collaborates with other digital control units within frequency-locked systems to build a complete digital frequency-locked system. The digital demodulation module can also obtain error signals with a high signal-to-noise ratio. This approach features high integration, flexibility, and outstanding stability, making it a key development direction in modern laser frequency-stabilization technology.

### 3.3. PID Control Algorithm

The PID control algorithm is a foundational closed-loop feedback method extensively used in laser frequency stabilization, valued for its structural simplicity, adjustable parameters, and robustness. PID control operates by continuously computing an error value as the difference between a desired setpoint and a measured process variable, then applying a correction based on proportional, integral, and derivative terms. The output of a continuous-time PID controller is typically expressed as:

(1) $$u\left(t\right)=K_{p}e\left(t\right)+K_{i}\int_{0}^{t}e\left(\tau \right)\;d\tau +K_{d}\frac{de(t)}{dt},$$

where e(t) represents the system error signal, and $K_{p}$, $K_{i}$, $K_{d}$ denote the proportional, integral, and derivative coefficients, respectively. The proportional term responds directly to the instantaneous error, improving the system’s reaction speed. The integral term eliminates steady-state errors, ensuring long-term stability. The derivative term anticipates the trend of error variation, enhancing dynamic performance. Appropriately tuned parameters enable the system to achieve both fast response and high steady-state accuracy. In the actual implementation on an FPGA, however, this continuous-time algorithm must be discretized and converted into a difference equation form for execution within digital logic.

In laser frequency stabilization systems, a PID controller is typically employed to adjust the laser drive current—thereby tuning its output frequency—or to generate a control signal for a PZT to modify the cavity length, compensating for frequency drift induced by temperature fluctuations, vibration, or power supply noise. When implemented on an FPGA, the PID algorithm can be executed in hardware logic with full parallelism, delivering control bandwidth sufficient for most stabilization requirements. Compared with conventional analog servo controllers, FPGA-based digital PID offers advantages such as programmable parameters and a reconfigurable structure, enabling the implementation of more sophisticated controller architectures (e.g., PI^3^) without hardware modifications and facilitating rapid iterative optimization of control performance during experiments [51,52]. Moreover, in the feedback control loop of laser frequency stabilization, the primary objective is often to suppress small-signal perturbations and eliminate steady-state error rather than to maximize response speed. Therefore, a proportional-integral (PI) controller is frequently adopted in practice. This avoids the introduction of a derivative term, which tends to amplify high-frequency noise and could otherwise degrade the stability of the locked system [53].

### 3.4. Intelligent State Monitoring and System Control

Beyond real-time signal acquisition and feedback control, FPGA-based laser frequency stabilization systems integrate intelligent state control logic to autonomously adapt to environmental variations and suppress long-term frequency drift. This functionality facilitates automatic switching between operational modes, thereby improving the system’s long-term stability and overall automation.

Before frequency stabilization, the system typically requires frequency scanning to determine the position of the reference signal or resonance curve, thereby locating the locking point where the error signal crosses zero. The FPGA can control the DDS module to generate a triangular wave or a sawtooth wave. After DAC conversion, this wave drives the piezoelectric ceramic to scan the laser frequency while simultaneously acquiring the photoelectric signal in real time to identify the locking point.

Once the lock point is identified, the system immediately transitions to the locked state. The internal PID controller within the FPGA begins operation. Leveraging the FPGA’s parallel processing advantages, multiple PID controllers can even run concurrently to meet complex multi-loop control requirements [54]. They continuously compute the deviation between the error signal and the reference frequency, outputting corresponding feedback control quantities to adjust the laser or piezoelectric ceramic, thereby stabilizing the frequency at the reference value.

The system may lose lock due to external disturbances such as vibration or acoustic noise. The FPGA can continuously monitor parameters such as the amplitude, derivative, or signal-to-noise ratio of the error signal. By implementing appropriate state identification algorithms [55], once loss of lock is detected, the system exits the locked state and initiates an automatic re-locking procedure, restarting the scanning and locking sequence to restore laser frequency stabilization at the reference frequency.

## 4. Evolution of FPGA-Based Laser Frequency Stabilization Architectures

Early laser frequency stabilization techniques, such as the PDH method and saturation absorption spectroscopy, were primarily implemented with analog circuits. These systems achieved frequency stabilization through precise analog electronics but suffered from limited hardware flexibility, restricting their adaptability to varying experimental conditions and impeding the integration of advanced control algorithms. The adoption of FPGAs ushered in a digital transition in this field. Leveraging high-speed parallel processing, real-time data acquisition, and reconfigurable logic, FPGAs facilitate the implementation of sophisticated signal processing and control functions that were previously unfeasible in analog frameworks.

### 4.1. FPGA as a Coprocessor

With the advancement of digital signal processing (DSP) technology, researchers first attempted to replace some analog modules with digital circuits [56,57,58]. In 2012, Bian et al. [59] reported a DSP-based PDH system. This system employed a DDS to generate modulated signals and utilized an analog mixer to demodulate the laser’s frequency drift information. Core control was performed by the DSP, achieving a relative frequency drift of no more than ± 17 kHz within 2.5 h. Its root mean square (RMS) error was 5 kHz, with absolute frequency stability better than 200 kHz.

In early digital implementations, the FPGA frequently served as a coprocessor dedicated to executing specialized algorithms. For instance, in 2016, Spindeldreier et al. [60] demonstrated an automated frequency estimation architecture built on an FPGA platform. In their design, the FPGA performed real-time frequency estimation on the output signal, compared it with the target frequency, and generated corresponding feedback control signals. Three correlation-based matching algorithms—Sum of Absolute Differences (SAD), Sum of Squared Differences (SSD), and Cross Correlation (CC)—were implemented and evaluated for frequency estimation. The system supported scalable performance through a configurable number of matching cores, ranging from 1 to 512. Among the three algorithms, SAD achieved the best overall performance in terms of matching rate, estimation error, FPGA resource utilization, and power consumption, confirming its suitability for this application.

**Figure 6:**
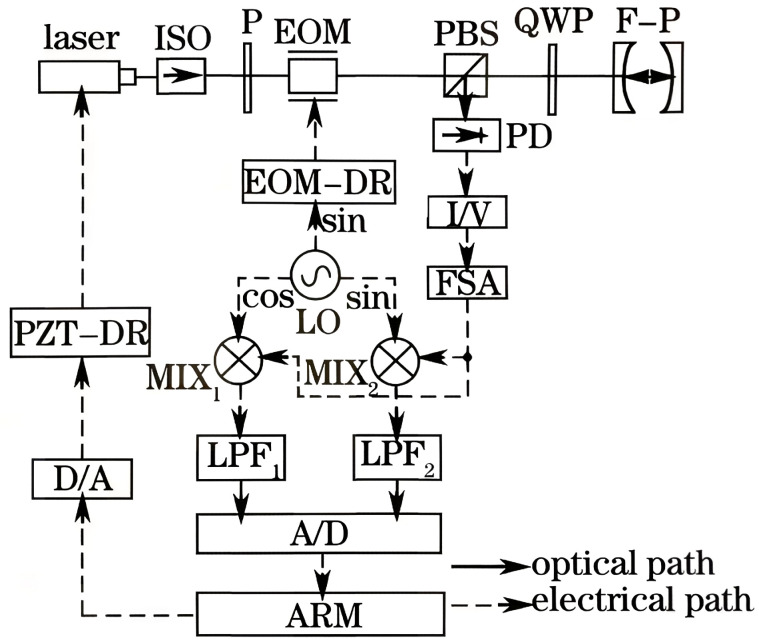
PDH frequency stabilization scheme based on quadrature demodulation [62]. Reproduced with permission from Su, J., Design of Pound–Drever–Hall laser frequency stabilization system using quadrature demodulation; published by Chin. J. Lasers, 2016.

As a coprocessor, the FPGA is also frequently integrated with analog circuits to form hybrid feedback loops. In 2015, Leibrandt et al. [61] presented a general-purpose digital servo system for laser feedback control in atomic, molecular, and optical physics experiments. Employing the PDH technique, they stabilized the frequency of a 1070 nm fiber laser to a Fabry--Perot (F-P) cavity. In this system, front-end operations such as demodulation were handled by analog components, while the error-signal processing and feedback-signal generation were implemented on the FPGA. The design also incorporated an automatic re-locking module based on threshold criteria. In 2016, Su et al. [62] proposed a PDH frequency-stabilization scheme based on quadrature demodulation, as illustrated in Figure 6. Their approach used a DDS to simultaneously generate multiple synchronous sinusoidal signals, enabling digital quadrature demodulation and thereby reducing phase errors and drifts inherent in analog mixing. This successfully avoided the phase-shifter errors and mixer DC-offset-induced signal distortion common in conventional PDH systems. Nevertheless, the generation and conditioning of the PDH error signal still relied primarily on analog circuitry. Although a digital PID algorithm was introduced, the overall architecture remained representative of a mixed analog-digital frequency-stabilization design.

### 4.2. All-Digital Frequency Stabilization System Based on FPGA

With continuous improvements in the logic resources, DSP units, and high-speed ADC/DAC interfaces of FPGA devices, laser frequency stabilization systems have progressively evolved from mixed-signal architectures toward all-digital architectures [63,64,65,66]. In an all-digital frequency stabilization system, the photodetector signal is directly sampled, and functions such as signal generation, mixing/demodulation, filtering, and PID control are implemented within the FPGA using hardware description languages, ultimately generating the feedback signal. All signal processing is performed in the digital domain [67,68]. This architecture completely eliminates DC drift and temperature-induced drift associated with analog mixers and filters, while offering exceptional flexibility and reconfigurability.

The advantage of the all-digital architecture lies in its powerful parallel processing capability. Traditional multi-frequency laser stabilization typically requires multiple independent analog electronic devices, which are difficult to synchronize. In 2013, Xing et al. [69] designed a dual-channel digital PDH frequency-locked system. The system employs a high-precision F-P cavity as the frequency reference. Using a single FPGA, two independent heterodyne interferometer subsystems were constructed to acquire dual electrical error signals. After digital signal processing within the FPGA (including digital low-pass filtering, phase detection, and PID control), the piezoelectric ceramics on the laser were driven to achieve simultaneous dual-frequency stabilization to the two resonant frequencies of the F-P cavity. This ultimately yielded frequency stability exceeding $1\times 10^{-10}$.

A further key benefit of all-digital architectures lies in the ability to implement advanced automated control sequences by utilizing the ample logic resources available within an FPGA. In 2015, Liu et al. [70] developed an all-digital frequency stabilization system for an external-cavity diode laser using LabVIEW FPGA. This system realized a complete digital workflow encompassing saturation absorption spectrum acquisition, automatic frequency scanning, spectral peak identification, error-signal demodulation, and PI feedback control. By programming an automated control sequence on the FPGA, the system could autonomously identify the saturation absorption peak during scanning to determine the lock point. During locked operation, it continuously monitored the error-signal status and initiated an automatic re-locking procedure upon detecting deviations, thereby significantly improving long-term system reliability.

**Figure 7:**
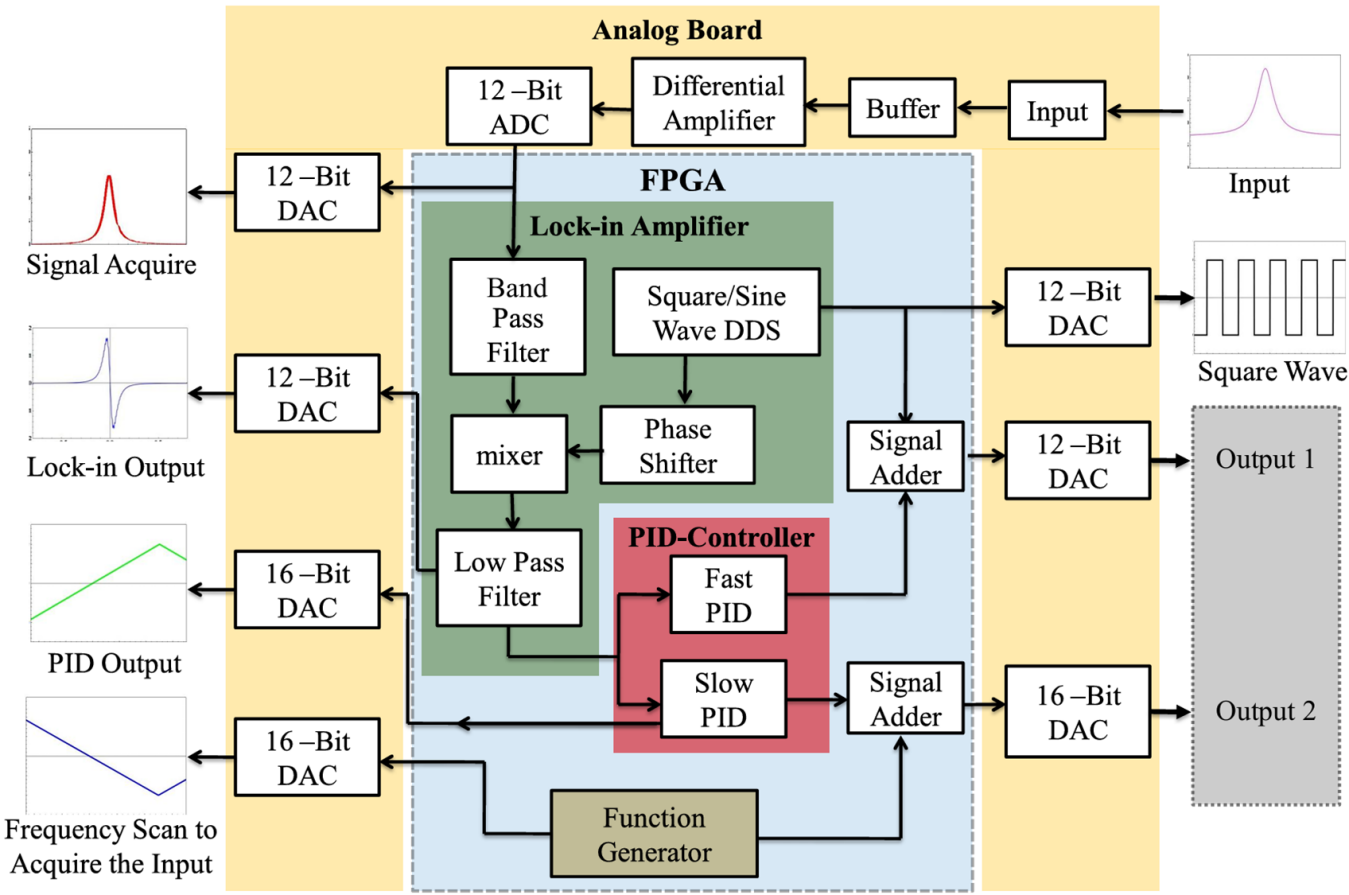
Low-cost digital control instrument for laser frequency stabilization based on a single-chip FPGA [71]. Reproduced with permission from Roy, A., An FPGA-based all-in-one function generator, lock-in amplifier and auto-relockable PID system; published by J. Instrum, 2019.

In 2019, Roy et al. [71] reported the automation of frequency stabilization systems by designing a low-cost digital control instrument based on a single-chip FPGA (Digilent CMOD A7) for laser frequency stabilization, whose schematic is depicted in Figure 7. Their implementation fully integrates a signal generator, a digital lock-in amplifier, and a PID controller within the FPGA while also incorporating an automatic re-locking mechanism. This mechanism monitors the error signal continuously; upon detecting loss of lock, the system automatically switches to scanning mode, scans the laser frequency, and reactivates the PID controller once the signal is reacquired, achieving a rapid recovery time of approximately 135 μs. Although the closed-loop bandwidth was constrained to 100 kHz by the hardware ADC, this work successfully illustrates the strong potential of all-digital platforms in executing complex control logic, improving system robustness, and lowering instrumentation costs.

In precision measurement fields that demand extremely high frequency stability, all-digital frequency stabilization systems have also demonstrated feasibility at ultra-high stability levels. Conventional analog PID controllers often suffer from integral windup when facing large disturbances or during the stabilization process, increasing tuning difficulty and impairing system recovery. In 2019, Didier et al. [72], in their 946 nm Nd:YAG laser frequency stabilization system, utilized an FPGA to implement the PDH error-signal generation and feedback control chain, replacing traditional analog mixers, filters, and feedback controllers. They programmed an 11.9 MHz quadrature lock-in amplifier inside the FPGA. To mitigate integral windup and simplify control-parameter optimization, the work introduced an integral-gain limiting algorithm into the digital PID controller, with the maximum integral gain given by:

(2) $$K_{i,\max}=\frac{K_{i}}{1-K_{\lim}}.$$

This design significantly simplified the optimization of PID parameters and effectively enhanced the control robustness during the stabilization process. Under long-term stable operation, the system achieved a frequency instability of $1.1\times 10^{-16}$ at an integration time of 1 s.

### 4.3. Intelligent and Integrated Systems Based on FPGA

With the trend toward unattended laboratory operation [73], higher demands are placed on the integration level, intelligence, and remote interaction capabilities of control systems. Conventional single-function firmware logic is no longer sufficient to meet these complex requirements. In response, an intelligent and integrated frequency stabilization system architecture based on the FPGA has emerged, marking a further evolution of laser frequency stabilization systems from all-digital toward intelligent operation.

Compared to all-digital frequency stabilization systems that prioritize high-speed signal processing and real-time feedback, intelligent frequency stabilization systems emphasize functional partitioning and system-level coordination. Typically built on an FPGA-SoC architecture, these systems allocate timing-critical and high-bandwidth tasks—such as signal acquisition, digital demodulation, and feedback control—to the programmable logic (PL) section. Meanwhile, functions including parameter management, operational scheduling, human–machine interaction, network communication, and advanced control algorithms are executed on the processing system (PS) side. Through hardware–software co-design, such systems achieve not only high control performance but also improved scalability and reliability.

The SoC architecture enables frequency stabilization systems to implement advanced operational logic and adaptive strategies without compromising feedback control performance. In 2018, Spindeldreier et al. [74] demonstrated a frequency modulation spectroscopy stabilization system built on an FPGA-SoC platform using a DFB diode laser. Their design integrated on-chip modules for signal demodulation, digital filtering, frequency estimation, and PI control within the FPGA. Additionally, a Sum-of-Absolute-Differences pattern-matching algorithm was embedded in the FPGA to perform absolute frequency estimation, thereby eliminating manual selection of atomic transition lines. Interactive control was managed by an embedded LEON3 processor, which supported remote configuration of filter parameters, PI settings, and stabilization operations via Ethernet. Experimentally, the system achieved a closed-loop control cycle of 82.08 μs. With a target frequency set to −586.6 MHz, the maximum residual frequency deviation after stabilization remained within approximately 12.0 MHz.

#### 4.3.1. Integrated Frequency Stabilization System Based on the Red Pitaya Platform

Among the various FPGA-SoC platforms, the open-source FPGA-based hardware platform Red Pitaya (STEMlab) is one of the earliest systems adopted for constructing low-cost digital frequency stabilization solutions [26]. Built on a Xilinx Zynq SoC, Red Pitaya integrates an FPGA with an embedded processor in a single device and provides high-speed ADCs/DACs, programmable digital I/Os, and network interfaces. This enables the platform to perform signal acquisition, digital processing, feedback control, and remote interaction on a single hardware unit, thereby addressing the limitations of traditional commercial frequency stabilization instruments, which are often costly and lack flexibility and scalability. Applications in laser frequency stabilization began to emerge around 2018 [75,76], with Red Pitaya being used to replace conventional analog modulators, mixers, and lock-in amplifiers, finding widespread use in PDH, saturation absorption, and related laser stabilization techniques [77].

In laser frequency stabilization systems [78], the Red Pitaya platform performs three core functions. Its onboard DACs output internally generated scanning or modulation signals to scan or phase modulate the laser frequency. The integrated FPGA executes digital mixing and demodulation, producing error signals for methods such as PDH and frequency modulation spectroscopy. Furthermore, the programmable logic implements high-speed digital PID control and feedback output, enabling high control bandwidth while lowering system complexity and cost. As a result, a single Red Pitaya board can replace multiple dedicated instruments typically required in conventional frequency stabilization setups.

**Figure 8:**
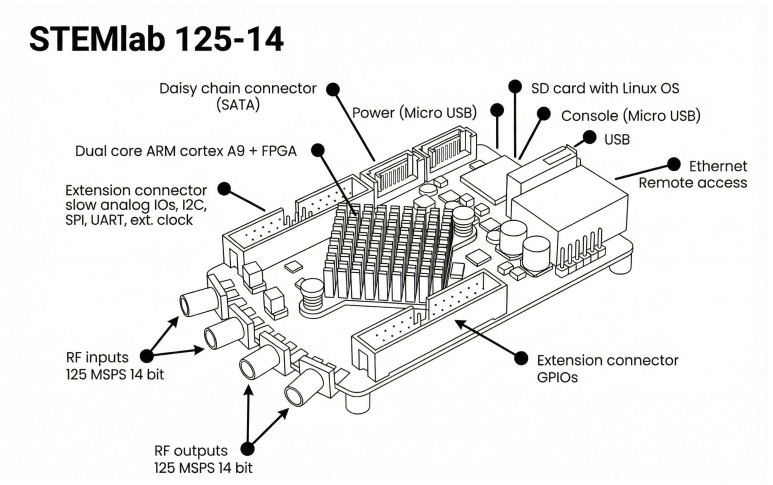
Red Pitaya board.

Currently, the most widely used version is the Red Pitaya STEMlab 125-14. A photograph of the actual unit is shown in Figure 8. It is equipped with two 125 MSps, 14-bit input channels and two 14-bit output channels, and is built on the Xilinx Zynq 7010 architecture. This integrates a dual-core ARM processing system with programmable logic in a single chip, providing both high-speed digital signal-processing capability and high system programmability [79] along with user-level operability. The system can be accessed via an online application interface over Ethernet or Wi-Fi.

#### 4.3.2. Open-Source Software Tools for Red Pitaya

Built around the Red Pitaya platform, researchers have developed various open-source software tools tailored for laser frequency stabilization applications. Representative tools include PyRPL, Linien, and lock-in+pid.

PyRPL [80] is an open-source digital feedback control platform designed for quantum optics and precision measurement applications. It integrates essential functions such as error-signal generation, digital filtering, PI control, a spectrum analyzer, and an oscilloscope. By allowing flexible configuration of control chains without FPGA re-synthesis, PyRPL supports diverse laser frequency stabilization tasks including PDH stabilization and cavity stabilization. In 2024, Zhang et al. [81] implemented a semiconductor laser frequency stabilization system on a Red Pitaya hardware platform using PyRPL. On a single FPGA board, they configured multiple functional modules—including an arbitrary waveform generator, a lock-in amplifier, a PID controller, and an oscilloscope—and successfully locked an external-cavity semiconductor laser to the saturation absorption spectrum of the cesium D_2_ line. The system demonstrated a frequency stability of 3.0 MHz over an 80-s measurement period.

Linien [82] is a spectroscopic locking platform whose FPGA firmware integrates an automated locking algorithm that compensates for signal jitter, allowing the system to identify and acquire the target lock point even under significant laser noise or unstable scanning conditions. Additionally, Linien incorporates a machine learning optimization module based on the Covariance Matrix Adaptation Evolution Strategy (CMA ES), which automatically adjusts parameters such as modulation frequency and depth to maximize the error signal slope, thereby improving locking robustness and long-term stability. The platform offers a complete control workflow that includes scanning, modulation, locking, and automatic re-locking, making it well suited for saturation absorption spectroscopy and other applications requiring unattended operation.

Lock-in+pid [83] is a web-based open-source toolkit designed for lightweight, cross-platform remote control through interaction between a web server and FPGA logic. It integrates a digital lock-in amplifier with an exceptionally wide operating frequency range, supporting square-wave demodulation up to 31.25MHz. The toolkit also features a deeply optimized PID controller that uses shift registers to enable parameter tuning across several orders of magnitude. Its locking control module allows automatic switching from scanning to locked mode based on signal level or timing triggers, along with automatic re-locking upon lock loss. Furthermore, the platform supports running resident programs on its onboard processor for continuous long-term data acquisition, enabling computation of the Allan variance to evaluate system frequency stability.

From a functional standpoint, PyRPL provides broad versatility and modular control suitable for diverse optical experiments. Linien specializes in spectroscopic locking with integrated parameter self-optimization, targeting unattended operation. Lock-in+pid excels in applications requiring high frequency modulation and demodulation capabilities.

## 5. Recent Research Progress

In recent years, advancements in FPGA performance, the growing maturity of FPGA-SoC architectures, and the expansion of open-source hardware and software systems have enabled FPGA-based laser frequency stabilization to evolve from merely replacing traditional analog circuits toward intelligent control and advanced digital signal processing. Global research progress confirms that the FPGA now serves as a core platform for implementing complex logic control, improving locking robustness, and building distributed frequency stabilization systems.

### 5.1. Intelligent Control and Fully Automatic Stabilization Technology

Conventional laser frequency stabilization systems frequently exhibit limited automation and robustness, often requiring manual intervention to maintain lock under significant environmental perturbations. Recent research indicates that embedding intelligent locking algorithms within the FPGA provides an effective solution. In 2025, Yu et al. [84] employed a custom FPGA board (model XC7K325) to fully digitize the servo feedback control. Their implementation integrated an automatic peak finding algorithm based on backward difference, which identifies the main spectral peak by comparing the temporal widths of successive signal peaks and subsequently triggers control state switching. Using this system, a commercial Nd:YAG laser was locked to a high-finesse F-P cavity for 30 min, achieving a relative frequency drift of 2MHz. To enhance robustness in complex operating environments, in 2025, Xia et al. [85] implemented a multi-loop automatic stabilization system on a single FPGA, integrating PDH frequency stabilization, fiber-noise cancellation (FNC), and laser power stabilization (LPS). This system can lock either the laser carrier or a frequency-shifted sideband to a reference cavity and successfully re-lock within 10 s following an interruption. It completed over 30,000 automatic locking and re-locking cycles, substantially improving the long-term reliability of the ultra-stable laser. The measured frequency instability reached $1.5\times 10^{-15}$ in carrier-locking mode and $2.5\times 10^{-15}$ in offset-locking mode at 1 s integration time.

**Figure 9:**
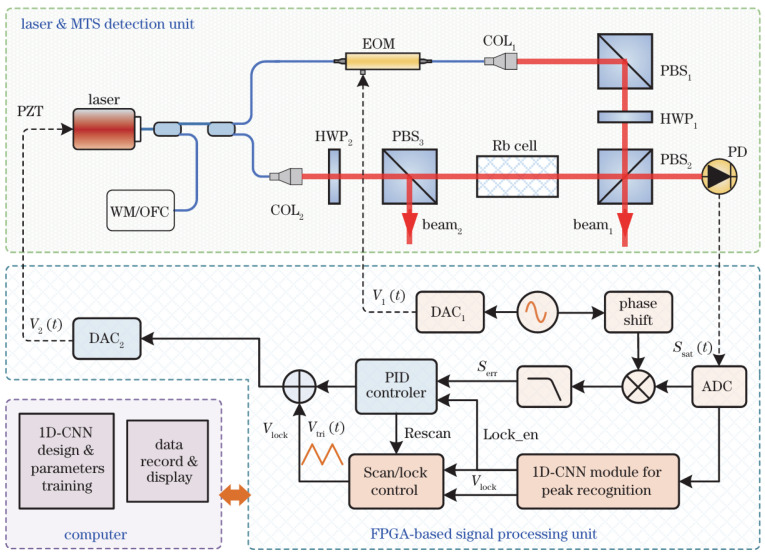
MTS laser frequency stabilization method for online identification of absorption peaks using FPGA-accelerated CNN [87]. Reproduced with permission from Yan, L., Laser frequency stabilization method based on FPGA-accelerated convolutional neural network absorption peak recognition (Invited); published by Laser Optoelectron. Prog, 2026.

Deep learning is increasingly being integrated into frequency stabilization systems to enhance intelligence and autonomy [86]. In 2025, Yan et al. [87] implemented an FPGA-accelerated CNN-based intelligent laser stabilization method for rubidium atomic saturation absorption peak recognition, with the experimental setup illustrated in Figure 9. Their optical system employed modulation transfer spectroscopy, and a lightweight 1D-CNN model was deployed on a Red Pitaya board to perform online recognition of the target absorption peak, automatic stabilization, and recovery after lock loss. Following stabilization, the laser frequency remained within 181.28 kHz over two hours, with a standard deviation of 26.89 kHz. The relative Allan variance reached $1.16\times 10^{-11}$ at an averaging time of 256 s.

### 5.2. Advanced Digital Signal Processing and Demodulation Algorithms

**Figure 10:**
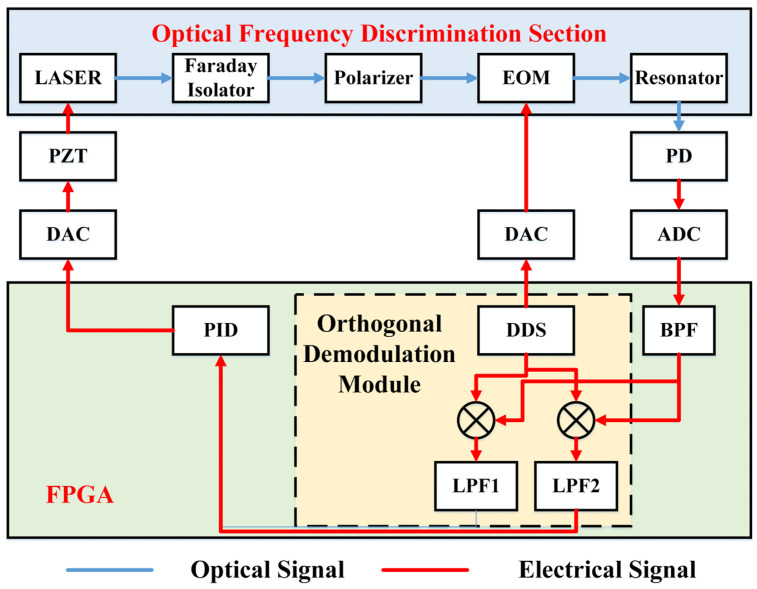
PDH system architecture implemented on the Harbin Institute of Technology FPGA platform [55]. Reproduced with permission from Liu, W., Optimization of the PDH frequency locking system based on the crystal birefringence theory and orthogonal demodulation scheme; published by Opt. Express, 2025.

Owing to its inherent advantages in high-speed digital signal processing and the ability to implement complex algorithms that are difficult to realize with analog circuitry, the FPGA can overcome the non-ideal characteristics of physical components, thereby improving the quality of error signals and enhancing system robustness. In 2025, LIU et al. from Harbin Institute of Technology [55], based on crystal birefringence theory, addressed the issue of phase instability in PDH system photodetector output signals caused by laser polarization deviation from the crystal optical axis and environmental disturbances—a problem that conventional demodulation schemes could not compensate for in real time. They proposed a novel quadrature demodulation scheme based on an FPGA, as shown in Figure 10. In this scheme, the root-mean-square (RMS) of sine and cosine signals is used instead of a single signal to generate the PDH error signal, effectively separating useful amplitude information from harmful phase noise. Additionally, a frequency-lock point positioning algorithm was developed, enabling precise localization of the locking point under complex environmental conditions and automatic re-locking after loss of lock.

Furthermore, addressing the trade-off between dynamic range and sensitivity in frequency stabilization systems, Yan et al. [79] proposed an adaptive PDH stabilization method in 2023 using a Red Pitaya development board combined with a dual-modulation-depth and dual-error-signal algorithm. The method first employs digital quadrature demodulation to achieve automatic matching of the demodulation phase, thereby improving the effective sensitivity of the PDH error signal. It further introduces a transmission-power signal to construct a pre-locking error signal with a large linear dynamic range. By combining different modulation depths, the system can switch between pre-locking and fine-locking modes. Additionally, the system can automatically switch operation modes based on changes in transmitted light intensity: maintaining lock or achieving fast recovery under strong disturbances, while ensuring high-precision stabilization under steady-state conditions. This approach extends the linear dynamic range of the system and achieves long-term relative stability on the order of $10^{-9}$.

### 5.3. Low-Latency Digital Servo Architectures and Latency Budget

Low latency is a key requirement for extending the feedback bandwidth of FPGA-based laser frequency stabilization systems. In a digital feedback loop, the total delay is not determined by the FPGA logic alone, but by the combined contributions of ADC conversion, FPGA data-path processing, digital demodulation and filtering, DAC reconstruction, analog output filtering, actuator response, and the optical plant. From a control-theory perspective, a pure time delay τ introduces an additional phase lag of approximately Δϕ≈−2πfτ at Fourier frequency *f*. Therefore, the unity-gain frequency is generally kept well below 1/(4τ) to preserve phase margin, although the exact stability boundary also depends on the actuator and plant transfer functions.

Several representative FPGA-based digital servo platforms have explicitly addressed this latency-bandwidth trade-off. Leibrandt and Heidecker reported an open-source custom FPGA servo for atomic, molecular, and optical physics experiments, providing an early general-purpose reference architecture with automatic lock acquisition, a total latency of 320 ns, and feedback bandwidths up to approximately 1 MHz [61]. Yu et al. used Terasic Altera DE2/DE3 development boards to benchmark low- and mid-range FPGA servo implementations against a commercial analog servo, demonstrating feedback bandwidths up to 2.5 MHz with servo signal latency below 200 ns, while the AOM delay of about 400 ns became the dominant delay in their test loop [51]. Pomponio et al. demonstrated a Koheron Alpha250-based low-latency digital servo as a compact FPGA/SoC implementation, reporting ADC, FPGA, and DAC delay contributions of 24 ns, 144 ns, and 32 ns, respectively, with a 1 MHz closed-loop demonstration [64]. More recently, Liu et al. developed a custom low-noise FPGA digital servo for ultra-stable laser frequency stabilization, reducing the total latency to 120.5 ns and achieving a closed-loop bandwidth of about 549.5 kHz, with a servo-noise contribution of only $5.54\times 10^{-18}$ at 1 s [88]. In contrast, Preuschoff et al. demonstrated the use of the commercial open-source STEMlab/Red Pitaya platform for compact and reconfigurable frequency and intensity stabilization, based on 14-bit ADC/DAC resources, with a measured open-loop delay of 200(5) ns and a controller bandwidth of 1.25 MHz [26].

Table 1 compares representative FPGA-based digital servo implementations in terms of reported latency, bandwidth, stability/noise performance, ADC/DAC configuration, and reported implementation-level features. The comparison makes one point clear: custom low-latency designs can push controller delay into the 100–300 ns range, whereas more general-purpose or low-cost platforms often trade some bandwidth for integration, software accessibility, and ease of use. The converter and implementation-feature columns further indicate that practical servo performance depends not only on the FPGA platform, but also on the surrounding mixed-signal hardware, firmware architecture, control interface, and automation functions. Therefore, the preferred FPGA-based servo architecture should be selected with the actuator, target loop bandwidth, converter requirements, and desired system functions in mind.

### 5.4. System-Level Frequency Stabilization Implementation and Architectural Design

While intelligent control algorithms and digital signal processing continue to evolve, recent research has further extended the advantages of the FPGA to the overall architectural design of laser frequency stabilization systems. In 2024, Majumder et al. [89] proposed a low-cost scheme based on frequency modulation spectroscopy. By implementing scanning signal generation, digital lock-in amplification, and PID control within a Red Pitaya, they consolidated the traditional analog modules into a compact frequency stabilization unit costing under $500, achieving long-term frequency stability on the order of 1 MHz.

**Figure 11:**
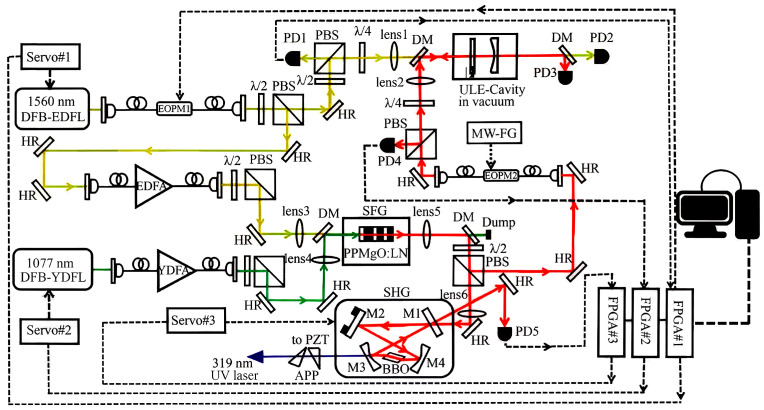
Distributed digital lock-in frequency system with multiple FPGAs working in concert [91]. Reproduced with permission from Su, W., Application of FPGA in frequency locking of a 319 nm ultraviolet single-frequency laser system; published by Acta Sin. Quantum Opt, 2025.

Distributed architectures offer distinct scalability and modular control advantages for complex optical systems. In 2023, Pultinevicius et al. presented a scalable scanning transfer-cavity frequency stabilization scheme based on a Red Pitaya platform [90]. Their implementation performed scanning-cavity transmission signal acquisition, peak detection, and error-signal generation on the device, successfully transferring the stability of a single reference laser to multiple target lasers with long-term frequency drift maintained below the MHz level. Further advancing this approach, Su et al. designed a multi-FPGA coordinated distributed digital frequency stabilization system in 2025 [91]. As shown in Figure 11, their experiment employed three independent Red Pitaya units to independently stabilize multiple wavelength lasers and a nonlinear frequency-doubling cavity concurrently. Through inter-unit coordination, the system achieved stable output of 319 nm ultraviolet resonance-enhanced light. This architecture significantly reduces experimental cost while offering high integration density and reconfigurability. More recent work has further extended FPGA-based frequency stabilization to specialized laser systems for Rydberg excitation. In 2026, Chang et al. [92] reported a Red Pitaya-based frequency stabilization scheme for a 509 nm single-frequency laser used in cesium Rydberg excitation. By comparing modulation-free Rydberg-TCPS and frequency-modulated Rydberg-EIT spectroscopy, they showed that the Red Pitaya platform could achieve stabilization performance comparable to that of conventional discrete instruments while offering lower cost and higher integration. This work indicates that FPGA-based system architectures are being increasingly applied to compact and application-specific laser stabilization systems in quantum optics and Rydberg-atom experiments.

## 6. Discussion and Conclusions

The FPGA-based laser frequency stabilization systems reviewed here differ in their physical references, error-signal generation mechanisms, feedback actuators, and digital architectures. Choosing among them is therefore not simply a matter of selecting the most advanced FPGA board. The required frequency stability, feedback bandwidth, wavelength reference, system complexity, cost, and level of automation all shape the appropriate stabilization strategy.

PDH locking is particularly suitable for applications requiring high short-term frequency stability and high-bandwidth feedback, such as ultra-stable lasers, optical clocks, precision spectroscopy, and cavity-stabilized systems. Its main advantages are the steep dispersive error signal and high sensitivity near cavity resonance. However, PDH systems usually require a stable optical cavity, phase modulation, careful control of residual amplitude modulation, and low-latency servo electronics. Therefore, PDH is generally advantageous over spectroscopy-based methods when an optical cavity with sufficiently high stability is available and when high feedback bandwidth is required.

Saturation-absorption-based methods provide absolute atomic or molecular frequency references and are attractive for compact atomic physics and spectroscopy systems. Direct saturation absorption has a relatively simple optical configuration, but its error-signal slope and background suppression can be limited. FMS shifts the detection to a modulation frequency and can reduce low-frequency technical noise, whereas MTS provides a background-suppressed dispersive signal with good long-term locking performance. These methods may be preferred when an absolute transition reference is more important than the highest possible servo bandwidth.

From the implementation perspective, mixed analog–digital architectures remain useful when low-noise analog front-end processing or high-bandwidth demodulation is required. In contrast, all-digital FPGA architectures offer stronger reconfigurability, compactness, multi-loop scalability, and automatic control. Custom FPGA servos can minimize loop latency and are suitable for demanding cavity-stabilized systems, whereas Red Pitaya provides a lower-cost and more accessible route for many laboratory applications. More recently, machine-learning-assisted and distributed FPGA systems have extended laser stabilization from single-loop feedback toward autonomous peak recognition, re-locking, and coordinated multi-channel control.

In summary, FPGA-based laser frequency stabilization is evolving from a dedicated digital signal-processing tool toward an integrated and intelligent control platform. Although FPGA systems provide strong flexibility, compactness, scalability, and automation, their ultimate performance is still constrained by ADC/DAC resolution, converter noise, loop latency, actuator bandwidth, quantization effects, and long-term robustness. Future developments are expected to focus on lower-latency digital servo architectures, higher-resolution mixed-signal interfaces, software-defined multi-loop control, and machine-learning-assisted autonomous operation. No single stabilization method is universally optimal; the best choice depends on the required stability, bandwidth, reference type, scalability, and automation level.

## Figures and Tables

**Table 1:** Comparison of representative FPGA-based digital servo platforms.

Authors	Platform Type	Reported Latency	Reported Bandwidth	Stability	ADC/DAC Configuration	System Features
Preuschoff et al. [26]	Commercial open-source STEMlab 125-14	200(5) ns open-loop delay	1.25 MHz controller bandwidth	52(1) kHz laser linewidth; intensity stability to $10^{-5}$ level	2 × 14-bit ADCs/DACs	Open-source; PyRPL
Yu et al. [51]	Commercial Terasic Altera DE2/DE3 boards	Total signal latency <200 ns; AOM delay ∼400 ns	Up to 2.5 MHz feedback bandwidth	Not reported	14-bit ADC/DAC pairs	Auto-lock; IIR filters
Leibrandt and Heidecker [61]	Custom-built servo box using an Opal Kelly XEM6010-LX150 module	320 ns total latency	Up to ∼1 MHz feedback bandwidth	Laser stability $2\times 10^{-15}$ for 0.5–10 s	Fast: 16-bit ADCs/DACs; slow: 20-bit DAC	MIMO; auto-lock
Pomponio et al. [64]	Commercial Koheron Alpha250 FPGA/SoC board	ADC 24 ns; FPGA 144 ns; DAC 32 ns	∼1 MHz closed-loop	Not reported	Main: 14/16-bit, 250 MSps; aux.: 24/16-bit, ∼40 kHz	Web/Python; auto-lock
Liu et al. [88]	Custom-made low-noise digital servo board based on a Xilinx Kintex-7 FPGA	120.5 ns total latency	549.5 kHz closed-loop bandwidth	Servo noise $5.54\times 10^{-18}$ at 1 s; laser stability $∼10^{-16}$ at 1 s	Dual 16-bit ADCs; fast/slow DACs	Custom; low-noise

## Data Availability

No new data were created or analyzed in this study. Data sharing is not applicable to this article.

## Abbreviations

The following abbreviations are used in this manuscript:

FPGA	Field-programmable gate array
PDH	Pound–Drever–Hall
SAS	Saturation absorption spectroscopy
FMS	Frequency modulation spectroscopy
MTS	Modulation transfer spectroscopy
DDS	Direct digital synthesis
ADC	Analog-to-digital converter
DAC	Digital-to-analog converter
PID	Proportional–integral–derivative
IIR	Infinite impulse response
EOM	Electro-optic modulator
PD	Photodetector
PZT	Piezoelectric transducer
